# DO DAILY STRESSOR TRAJECTORIES VARY BETWEEN CIVILIANS, NONCOMBAT, AND COMBAT VETERANS?

**DOI:** 10.1093/geroni/igae098.0942

**Published:** 2024-12-31

**Authors:** Maria Kurth, Dakota Witzel, David Almeida

**Affiliations:** The Pennsylvania State University, University Park, Pennsylvania, United States; South Dakota State University, Brookings, South Dakota, United States; Pennsylvania State University, University Park, Pennsylvania, United States

## Abstract

Proximal (i.e., daily) experiences may be a crucial indicator of how larger-scale events, such as trauma, impact health and well-being (Epel et al., 2018). It has also been suggested that military service – especially combat exposure – is a “hidden” variable of aging (Spiro et al., 2016), with positive and negative long-term consequences for well-being. However, few studies have directly compared differences in civilian and Veterans’ daily experiences, especially exposure to stress. We used data from three waves of the National Study of Daily Experiences (NSDE; Almeida, 2005) to examine how daily stressor exposures change across ~20 years (N = 2220). At each wave, participants completed eight end-of-day phone interviews, including the Daily Inventory of Stressful Events (DISE; Almeida et al., 2002). For this project, we were interested in the following stressor domains: argument; avoided argument/disagreement; home; work/school. Similar to previous research (e.g., Piazza et al., 2022), civilian (83%) non-combat Veteran (12%), and combat Veteran status (5%) was gleaned from responses to two Life Event Survey items (LES; Sarason et al., 1978). We estimated two-level generalized multilevel models for each stressor domain and controlled for age, sex, education, and race. Preliminary results suggest only avoided argument exposure varied by civilian/Veteran status. Although there was no difference at baseline, civilians increased in likelihood (~20%), while combat Veterans were ~15% less likely to experience avoided arguments every 10 years. Results underscore a potential long-term advantage for aging combat Veterans’ well-being, as avoiding arguments has negative consequences for physical and psychological health.

